# von Willebrand Factor and Platelet Glycoprotein Ib: A Thromboinflammatory Axis in Stroke

**DOI:** 10.3389/fimmu.2019.02884

**Published:** 2019-12-17

**Authors:** Frederik Denorme, Karen Vanhoorelbeke, Simon F. De Meyer

**Affiliations:** Laboratory for Thrombosis Research, KU Leuven, Kortrijk, Belgium

**Keywords:** von Willebrand factor, platelet glycoprotein Ibα, thromboinflammation, ischemic stroke, ADAMTS13

## Abstract

von Willebrand factor (VWF) and platelets are key mediators of normal hemostasis. At sites of vascular injury, VWF recruits platelets via binding to the platelet receptor glycoprotein Ibα (GPIbα). Over the past decades, it has become clear that many hemostatic factors, including VWF and platelets, are also involved in inflammatory processes, forming intriguing links between hemostasis, thrombosis, and inflammation. The so-called “thrombo-inflammatory” nature of the VWF-platelet axis becomes increasingly recognized in different cardiovascular pathologies, making it a potential therapeutic target to interfere with both thrombosis and inflammation. In this review, we discuss the current evidence for the thrombo-inflammatory activity of VWF with a focus on the VWF-GPIbα axis and discuss its implications in the setting of ischemic stroke.

## von Willebrand Factor In Hemostasis: Recruitment Of Platelets

In this section, we briefly summarize the synthesis, structure, and role of VWF in hemostasis and refer to more extensive reviews for further reading.

**Figure 1:**
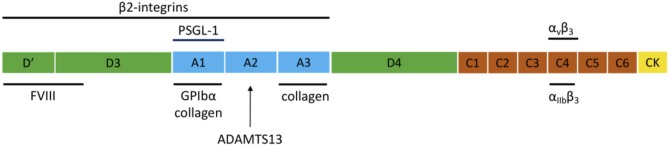
Domain structure of VWF and the main interaction sites. The domain structure of VWF is given and the most important interactions for inflammation and hemostasis are indicated.

VWF is a large multimeric plasma protein that plays a major role in hemostasis (1–4). First, VWF recruits platelets to sites of vascular injury by forming a bridge between the damaged vessel wall and platelets. Second, VWF also serves as a carrier protein for coagulation factor VIII (FVIII) and hence protects FVIII from degradation, cellular uptake or binding to the surface of activated platelets and endothelial cells (5). VWF is produced exclusively by endothelial cells and megakaryocytes. VWF is synthesized as a pre-pro-VWF that consists of a 22 amino acid signal peptide, a 741 amino acid propeptide (D1-D2) and a mature subunit of 2,050 amino acids (6). The mature subunit is composed of different types of domains arranged in the following order: D′-D3-A1-A2-A3-D4-C1-C2-C3-C4-C5-C6-CK (Figure 1) (1, 7).

After removal of the signal peptide, pro-VWF monomers dimerize in the endoplasmic reticulum through disulfide linkage of the C-terminal CK domains. In the Golgi complex, complete multimerization of the dimers occurs via disulfide linkage of the N-terminal D3 domains, together with additional modifications such as removal of the propeptide, glycosylation, and sulfation. After synthesis, VWF is either constitutively secreted into the blood or is stored in endothelial Weibel-Palade bodies (WPB) and platelet α-granules, from which VWF is locally released *via* regulated secretion (8). Basolateral release of endothelial VWF leads to accumulation of VWF in the subendothelial matrix, which becomes exposed following damage to the vessel wall. Ultra Large-VWF (UL-VWF) that is released at the apical surface can remain anchored on the surface of endothelial cells and form platelet-decorated strings (9). The structure of VWF is important for its function since several domains are essential for the hemostatic activity of VWF, such as the A1, A3, and C4 domains that mediate binding to GPIbα, collagen, and αIIbβ3, respectively (Figure 1).

The interaction of VWF with platelet GPIbα is crucial for initial platelet adhesion, especially in environments with high hemodynamic shear forces. GPIbα is a subunit of the platelet GPIb-IX-V complex that also contains the GPIbβ, GPIX, and GPV subunits, all of which are type I transmembrane proteins containing leucine-rich repeat domains. Under normal conditions, VWF circulates as a globular protein in which the binding site for GPIbα in the A1 domain is not accessible. However, upon blood vessel damage, VWF binds via its collagen binding sites (mainly in the A1 and A3 domains) to the exposed subendothelial matrix. Immobilization and flow shear forces then result in a conformational activation of the VWF A1 domain, enabling binding of the N-terminal domain of GPIbα (4). This force-induced regulation of the VWF-GPIbα interaction occurs via changes in intramolecular shielding of the VWF A1 domain by neighboring VWF sequences, possibly together with intrinsic changes in the affinity state of the VWF A1 domain itself (10).

The reversible nature of the VWF A1-GPIbα interaction permits platelets to roll and thus decelerate on immobilized VWF, ultimately allowing firm adhesion of platelets to the exposed subendothelial matrix via the platelet collagen receptors GPVI and integrin α2β1. The GPIbα-VWF and GPVI/α2β1-collagen interactions induce downstream intracellular platelet signaling leading to activation of platelet αIIbβ3, which mediates further stable adhesion and aggregation via binding to fibrinogen and VWF.

A central aspect of VWF activity is that larger VWF multimers are more active due to the presence of more monomeric subunits and the higher sensitivity for shear forces. UL-VWF multimers have a molecular weight of >10,000 kD and are highly reactive because the GPIbα binding sites in the VWF A1 domains are continuously exposed. As a result, spontaneous binding of platelets to VWF can occur. UL-VWF is stored in the endothelial WPBs from which it is released via both basal and regulated secretion pathways but also in platelet α-granules from which it is released only after agonist-induced stimulation (6). The local, regulated release of UL-VWF allows fast and confined hemostasis when needed at sites of injury. To prevent accumulation of prothrombotic UL-VWF, however, UL-VWF is cleaved by the VWF cleaving protease ADAMTS13 (A Disintegrin and Metalloprotease with ThromboSpondin type 1 repeats, number 13). Proteolysis of VWF by ADAMTS13 occurs in the VWF A2 domain and is dependent on conformational activation of the A2 domain to expose the cleavage site (11). Digestion of UL-VWF by ADAMTS13 results in smaller, less active VWF multimers (≤10,000 kDa) that adopt a folded conformation in which the platelet binding site in the A1 domain and the ADAMTS13 cleavage site in the A2 domain are cryptic. In the absence of ADAMTS13, spontaneous formation of VWF-platelet complexes leads to thrombotic complications as seen in patients with thrombotic thrombocytopenic purpura (12).

## von Willebrand Factor in Inflammation: Recruitment of Leukocytes

Besides its well-established role in hemostasis, VWF is recognized as an effective mediator of inflammatory responses as well. VWF can actively participate in the development of inflammatory processes by recruiting leukocytes at sites of vascular inflammation. Indeed, VWF deficiency or blockade has been shown to reduce leukocyte recruitment in various murine models of inflammation, including cytokine-induced meningitis (13), wound healing (13), atherosclerosis (14), cutaneous inflammation (15, 16), vasculitis (17), and peritonitis (18). When studying the inflammatory effects of VWF, it is important to keep in mind that VWF itself is essential for the formation of WPBs in endothelial cells (19). Alongside VWF, WPBs store also other molecules involved in inflammation and even angiogenesis (e.g., P-selectin, interleukin-6, interleukin-8, Eotaxin-3, Factor H, and angiopoietin-2). Failure of co-storage of inflammatory proteins in the endothelium of VWF-deficient mice can thus also cause defects in inflammation (20). However, recent research provided ample evidence for a direct role of VWF in inflammation, which might potentially be more important than co-storage of inflammatory proteins in the acute-phase response of the vessel wall.

When endothelial cells are activated by inflammatory mediators, UL-VWF is rapidly released from endothelial WPBs. As a consequence, increased levels of circulating VWF antigen has become a well-known marker of inflammation and endothelial activation. When secreted into the blood stream, released VWF can also remain anchored on the surface of endothelial cells through binding with P-selectin (21), integrin αVß3 (22), or the glycocalyx (23) and locally form platelet-decorated strings. VWF facilitates inflammatory processes by promoting leukocyte recruitment to inflamed tissues, either directly or indirectly after binding platelets.

An elegant study by Pendu et al. demonstrated that VWF can act as an adhesive surface for neutrophils and monocytes and that the adhesion process of these inflammatory cells involves various interactions that act in a concerted way (24). Direct adhesion of leukocytes occurs via multiple regions within the VWF molecule that interact with PSGL-1 and ß2 integrins on leukocytes (Figure 1). Whereas, PSGL-1 would be involved in initial rolling on VWF, ß2 integrins would be responsible for stable adhesion on VWF. ß2 integrins can interact with two distinct binding sites on VWF that are located in the D′D3 and A1-A2-A3 regions of VWF as well as to the Leu-Leu-Gly motifs found in the VWF D3 and the connecting region between the A1 and A2 domains (24, 25). The binding site for PSGL-1 is located in the VWF A1 domain (24). Similar for binding to GPIbα, the A1 domain needs to be in its active conformation to bind PSGL-1, which shares structural similarities with GPIbα (24).

Apart from binding directly to leukocytes, VWF can also indirectly promote leukocyte recruitment by forming VWF-platelet-leukocyte complexes, with a crucial role for the VWF-GPIbα axis. The exact mechanisms by which VWF-platelet complexes facilitate leukocyte diapedesis are not yet fully understood and might vary between different inflamed tissues. First, activated platelets bind to VWF and can then interact via P-selectin or GPIbα with leukocytes, thus promoting local adhesion of inflammatory cells (26). As such, immobilized VWF can function as a local matrix to recruit both platelets and leukocytes. Whereas, direct interactions between VWF and leukocytes might be sufficient under venous low-shear conditions, it is conceivable that platelets are needed for leukocyte recruitment under arterial high-shear conditions (26). Second, VWF-platelet complexes can regulate vascular permeability, leading to facilitated leukocyte extravasation. Indeed, using a model of thioglycollate-induced peritonitis, Petri et al. showed that leukocyte recruitment to the inflamed peritoneum was dependent on the presence of VWF and platelets and more specifically on the functional availability of GPIbα (18). In this study, the contribution of VWF-platelet complexes could be explained by destabilization of the endothelial barrier function rather than by increased leukocyte rolling and adhesion. The possible mechanisms through which VWF and platelets induce vascular leakage need further study. Endothelial permeability might be regulated by binding of the VWF RGD motif to endothelial αVß3 integrins (22), and platelets can release various soluble factors that influence endothelial junctions (27). A recent study also showed the involvement of microparticles in VWF-mediated vascular leakage (28).

Overall, the VWF A1 domain seems to be central for the participation of VWF in inflammatory processes. This was recently underlined by two studies from the group of Cécile Denis and Peter Lenting showing that specific inhibition of the VWF A1 domain leads to reduced vascular permeability and leukocyte recruitment (17), whereas a gain-of-function mutation in the VWF A1 domain results in increased leukocyte recruitment (16). Also clinically, the presence of an active A1 domain was shown to predict mortality in patients with systemic inflammatory response syndrome (29). Since an active A1 domain is a typical hallmark of UL-VWF in circulation, it might not be surprising that ADAMTS13 can exert anti-inflammatory activity by reducing the activity of VWF. By cleaving VWF, ADAMTS13 can remove VWF strings from the endothelial surface or reduce the size of reactive VWF to less adhesive VWF molecules. As a result, ADAMTS13 is able to attenuate VWF-dependent leukocyte rolling, adhesion, and extravasation under acute inflammatory conditions. The anti-inflammatory properties of ADAMTS13 have been demonstrated in various settings, including peritonitis (30), atherosclerosis (31), colitis (32), myocardial infarction (33–35), cardiac fibrosis (36), and ischemic stroke, as discussed further.

## VWF-Glycoprotein Ib Mediated Thromboinflammation in Ischemic Stroke

Ischemic stroke occurs when a blood clot obstructs cerebral blood flow and causes ischemic brain damage. The primary objective in acute ischemic stroke care is achieving fast reperfusion of the occluded blood vessel to limit ischemic brain injury. Yet, sometimes progressive stroke still develops despite reperfusion of the affected brain tissue, a phenomenon attributed to “reperfusion injury” (37, 38). It has become clear that cerebral ischemia/reperfusion injury is a complex pathology that involves crosstalk between both thrombotic and inflammatory pathways, which has lead to the concept of thrombo-inflammation in stroke (39, 40). Given the dual role of VWF and GPIbα in both thrombosis and inflammation, the VWF-GPIbα axis has received quite some attention in the setting of ischemic stroke (41).

Evidence for the involvement of VWF in ischemic brain injury comes from mouse studies showing that absence of VWF is associated with a significant reduction in ischemic stroke brain injury and improved functional outcome (42, 43). The detrimental effects of VWF were later attributed to the specific involvement of the VWF A1 and A3 (but not C4) domains, indicating a key role for the VWF-GPIbα and VWF-collagen interactions (44). Of note, whereas platelet-derived VWF is largely dispensable for normal hemostasis and thrombosis in mice, we showed that it can actively contribute to ischemic brain injury via a mechanism that is GPIbα-dependent (45). In parallel with these studies on VWF, similar research demonstrated that also GPIbα is an important mediator of cerebral ischemia/reperfusion injury. Indeed, mice lacking functional GPIbα also develop smaller brain infarctions together with improved stroke outcome (46, 47), an observation that was extended in a more translational setting using aged and comorbid (atherosclerotic, diabetic, and hypertensive) animals (48). Furthermore, anfibatide, a snake venom-derived GPIbα antagonist that specifically blocks platelet GPIbα binding to VWF had a potent protective effect in mouse models of ischemic stroke (49–52). As mentioned above, UL-VWF can spontaneously bind platelets and its reactivity can cause thrombotic events without proper regulation by ADAMTS13. Accordingly, experimental stroke studies showed that ADAMTS13-deficient mice developed larger brain infarctions and worse neurologic outcomes, whereas infusion of recombinant ADAMTS13 was able to attenuate ischemic brain damage (42, 53–56). Together, these studies highlight the pathophysiological involvement of VWF and GPIbα in cerebral ischemia/reperfusion injury, which can be counterbalanced by blocking the VWF-GPIbα interaction or by reducing the activity of VWF via ADAMTS13.

The precise mechanisms underlying the pathophysiological involvement of the VWF-GPIbα axis in ischemic brain injury are not yet fully elucidated but available data strongly points toward an intricate process that includes both thrombotic and inflammatory pathways. The cerebral microvasculature rapidly responds to brain ischemia leading to endothelial cell activation and endothelial denudation exposing subendothelial matrix components such as collagen. It has been long known that local platelet and leukocyte recruitment can lead to microvascular obstruction within the ischemic territory after occlusion and reperfusion, a process known as the “no-reflow” phenomenon (57–59). Given the fundamental role of VWF and GPIbα in thrombus development at sites of vascular damage, it is not surprising that the VWF-GPIbα axis is responsible for thrombotic events in stroke. In mouse models of cerebral ischemia/reperfusion injury, VWF deficient mice indeed showed less thrombosis in the cerebral microvasculature, as shown by reduced intracerebral fibrin(ogen) deposition in the affected brain tissue of these animals compared to wild-type mice (44, 45, 60). Remarkably, fibrin(ogen) deposition was considerably reduced in the ischemic hemisphere of the VWF deficient mice that were reconstituted with VWF defective in binding to fibrillar collagen or GPIbα compared with controls, again emphasizing the contribution of initial platelet adhesion interactions mediated by VWF (44). By specifically blocking the VWF-GPIbα axis, anfibatide reduced the number of fibrin(ogen)-positive blood vessels and microthrombi in the ischemic hemisphere (49, 51). In line with these results, anti-GPIbα treatment significantly reduced thrombus burden in the cerebral microvasculature, as measured by the number of GPIX-positive platelet aggregates and occluded brain vessels (61). Correspondingly, ADAMTS13 deficient mice showed an increased number of thrombi containing fibrin and VWF in the brain lesions after stroke (53). Recently, analogous observations were made in CD69 deficient mice (62). CD69 was identified as a negative regulator of endothelial VWF release, and in the setting of stroke, its absence resulted in a more severe stroke burden due to increased cerebral thrombosis (62).

Remarkably, whereas thrombus formation requires both platelet adhesion via GPIbα and GPVI and platelet aggregation via αIIbβ3, the latter does not seem to play a major role in acute ischemic stroke injury (44, 46, 48). Hence, platelets and VWF most likely contribute to stroke progression in a way that is not strictly related to thrombus formation. The most plausible explanation is the involvement of a corresponding inflammatory component mediated by the initial interactions between the damaged vessel wall, VWF, and platelets. Ample evidence for such an inflammatory reaction has been gathered in the last decade. Indeed, in mouse models of ischemic stroke, VWF deficiency is associated with reduced neutrophil infiltration in the ischemic hemisphere (55). In addition, expression levels of the pro-inflammatory cytokines IL-6, IL-1ß, and tumor necrosis factor-α are also decreased in the absence of VWF (55, 60). Interestingly, endothelial-derived rather than platelet-derived VWF seems to be the major determinant of these inflammatory effects (60). In line with the high activity of UL-VWF, elevated VWF-mediated inflammation is observed in the injured brain hemisphere of ADAMTS13-deficient mice. Increased myeloperoxidase activity, increased extravasation of neutrophils, and a higher expression of inflammatory cytokines high-mobility group box1, IL-6, and tumor necrosis factor-α were observed in ADAMTS13-deficient mice compared with wild-type controls (53–55). Interestingly, the increased brain damage and worsened neurological outcome observed in ADAMTS13-deficient animals were abrogated when neutrophils were depleted, indicating a causal role of neutrophils in the exacerbation of ischemic brain injury in the absence of ADAMTS13 (55). Blockade of GPIbα similarly led to decreased expression of IL-6, IL-1ß, and tumor necrosis factor-α (50, 61) and was also shown to lower the numbers of infiltrating T-cells and myeloid leukocytes (51, 61). The latter is in accordance with recent data from our group showing that inhibition of the VWF-GPIbα interaction results in significantly decreased recruitment of monocytes, neutrophils, and T-cells in the ischemic brain (63).

In summary, current evidence shows the involvement of the VWF-GPIbα interaction in a vicious circle of thrombotic and inflammatory responses in the ischemic stroke brain. Ischemia leads to endothelial damage, exposure of subendothelial matrix, upregulation of adhesion molecules, and release of UL-VWF. Local accumulation of VWF contributes to intravascular recruitment platelet and leukocytes, which can secrete proinflammatory cytokines that further stimulate inflammation. Aggregates of VWF, platelets, and leukocytes most probably plug brain capillaries, preventing efficient microcirculatory reperfusion. However, many aspects of the spatiotemporal involvement and molecular interactions between VWF and leukocytes in stroke remain to be elucidated. For, example, whether direct interactions between VWF and PSGL-1 and ß2 integrins are involved remains unanswered. Also, the potential effect of VWF and platelets on vascular permeability in the stroke brain needs further study. Initial results indeed indicate that interfering with VWF or GPIb can modulate the cerebrovascular integrity after stroke (51, 64). When blocking the function of GPIbα, it is important to realize that this platelet receptor contributes to arterial thrombosis *via* additional mechanisms that are independent of its binding to VWF (65). GPIbα also interacts with various other ligands such as thrombin, coagulation factors XI and XII, high molecular weight kininogen, and thrombospondin-1. Hence, further studies are needed to generate a more complete picture of the involvement of GPIbα in ischemic stroke, besides binding to VWF.

New insights show that already very early during ischemia, neutrophils, and platelets are recruited to the ischemic brain and contribute to microvascular dysfunction (66, 67). Otxoa-de-Amezaga and colleagues recently visualized an early influx of neutrophils to the brain after stroke, predominantly located within the intravascular space already early after reperfusion (68). It would be interesting to further untangle the specific role of VWF during these very early responses in the ischemic tissue to better understand the involvement of VWF in the neurovascular unit.

## Translational Aspects

The clinical significance of the VWF-GPIbα interaction in stroke is suggested by an increasing number of human stroke studies showing the pathophysiological involvement of VWF in ischemic stroke (69–74). Furthermore, polymorphisms in the GPIBA gene that lead to enhanced VWF-GPIbα interactions are associated with an increased risk of ischemic stroke in humans (75). Intriguingly, increased VWF activity and/or reduced ADAMTS13 activity are associated not only with higher stroke occurrence, but also with worse long-term stroke outcomes (71, 76–78). Nonetheless, more clinical studies are needed to specifically address the contribution of VWF-mediated thromboinflammatory brain damage during ischemia and reperfusion in ischemic stroke patients. From a clinical perspective, it is promising that the first-generation of VWF-inhibitors is currently enrolled in clinical studies for thrombotic thrombocytopenic purpura, such as a specific inhibitor of the VWF-GPIbα interaction (79) and recombinant ADAMTS13 (80). Notably, we and others have demonstrated that targeting VWF can also promote blood clot dissolution in the setting of ischemic stroke (81–85), which could be of particular relevance to overcome thrombolysis resistance of platelet-rich blood clots in patients (86). Hence, compounds that target VWF could have the attractive potential to promote acute thrombolysis in the occluded blood vessel and attenuate ischemia/reperfusion injury in the microvasculature of the affected brain territory. The safety, especially in terms of bleedings, remains to be further investigated before clinical use. At least in preclinical animal research, targeting VWF via anti-VWF-GPIbα strategies or recombinant ADAMTS13 did not increase the risk of intracranial hemorrhaging in murine stroke models (42, 43, 49), even when combined with tissue-plasminogen activator (87, 88) or when treatment was delayed (56). Of note, ADAMTS13 therapy improved outcomes in murine models of intracerebral hemorrhage in a VWF-dependent way (89–92). More research, preferably also in larger animal models, is needed to bring the concept of blocking VWF-mediated thromboinflammation in stroke closer to the clinic.

## Author Contributions

All authors listed have made a substantial, direct and intellectual contribution to the work, and approved it for publication.

### Conflict of Interest

The authors declare that the research was conducted in the absence of any commercial or financial relationships that could be construed as a potential conflict of interest.
